# Editorial Comment: The protective effect of Papaverine and Alprostadil in rat testes after ischemia and reperfusion injury

**DOI:** 10.1590/S1677-5538.IBJU.2017.0600.1

**Published:** 2018

**Authors:** Fernando Lorenzini

**Affiliations:** 1 Centro de Reprodução Humana Curitiba, PR, Brasil

In testicular torsion (TT), not only the ischemia due to TT but also the reperfusion during detorsion, both phenomena lead to testicular damages, so-called ischemia/reperfusion (I/R) injuries. Many drugs can help to protect these injuries, specially the antioxidants.

The authors in this article (1), using Cosentino score (CS) and Johnson tubular biopsy score (JTBS) to analyze the I/R testicular injuries, in rats submitted to TT maintained for four hours and applying papaverine or alprostadil directly to the spermatic cord during the surgical detorsion, demonstrated the protective effects of both drugs on the I/R injuries. JTBS demonstrated that both drugs had significant protective effects, but CS demonstrated that only alprostadil showed statistically significant protective effects. So, alprostadil seems to be better than papaverine for this purpose.

In the clinical practice, there is a need for more studies in order to accept, as a guideline, the use of the alprostadil or papaverine as having protective effects on testicular I/R injuries.
